# Cigarette smoke exposure induces expression of the pneumococcal erm(B) macrolide resistance gene

**DOI:** 10.18332/tid/113528

**Published:** 2019-11-19

**Authors:** Kgashane Given Matapa, Therese Dix-Peek, Riana Cockeran, Ronald Anderson, Helen C. Steel, Charles Feldman

**Affiliations:** 1 Department of Medical Immunology, University of Pretoria, Pretoria, South Africa; 2 Institute for Cellular and Molecular Medicine, University of Pretoria, Pretoria, South Africa; 3 Clinical Haematology Division, University of the Witwatersrand, Johannesburg, South Africa; 4 Division of Pulmonology, University of the Witwatersrand, Johannesburg, South Africa

**Keywords:** antibiotic resistance, macrolides, pneumococcus, smoking, streptococcus pneumoniae

## Abstract

**INTRODUCTION:**

Cigarette smoking is a well-recognized risk factor for development of severe, invasive pneumococcal disease. However, little is known about the direct effects of exposure to cigarette smoke on the virulence mechanisms of the pathogen, particularly in respect of resistance to macrolide antibiotics, which are widely used in the treatment of pneumococcal infection. This study aimed to investigate the effects of exposure to cigarette smoke condensate (CSC, 80 and 160 mg/L) and clarithromycin (2 and 8 mg/L), alone and in combination *in vitro*, on expression of the *erm*(B) and *mef*(A) macrolide resistance genes of strains 2507 and 521 (both serotype 23F), respectively, of the pneumococcus.

**METHODS:**

Following exposure to CSC or clarithromycin, individually and in combination, *erm*(B) and *mef*(A) gene expression were measured by sequential extraction of RNA, conversion to and amplification of cDNA, and detection by qRT-PCR.

**RESULTS:**

As expected, exposure of both test strains of the pneumococcus to clarithromycin resulted in substantial upregulation of both macrolide resistance genes, which was significantly (p<0.001) augmented by prior exposure to CSC in the case of *erm*(B), but not *mef*(A). Somewhat unexpectedly, exposure of strain 2507 to CSC (160 mg/L) alone (in the absence of clarithromycin) also resulted in significant (p<0.05) expression of the *erm*(B) gene.

**CONCLUSION:**

Although the possible clinical significance remains to be established, these findings suggest that smoking may impede the efficacy of macrolide-based antimicrobial therapy by accelerating the onset and magnitude of *erm*(B)-mediated resistance, representing a novel pro-infective mechanism of smoking.

## INTRODUCTION

Globally, the burden of disease associated with *Streptococcus pneumoniae* (pneumococcus) infections is considerable, a situation which is compounded by the fact that treatment of pneumococcal infection has become increasingly challenging due to the growing threat of antibiotic resistance^1^. Cigarette smoking, still prevalent in many countries, is recognized as being one of the most prominent independent risk factors for invasive pneumococcal disease^2^. Moreover, current smokers who develop pneumococcal community-acquired pneumonia (CAP) have a striking 5-fold increase in the risk of 30-day mortality irrespective of age, co-morbidities and early implementation of guideline-concordant antimicrobial chemotherapy^3^.

Tobacco-induced susceptibility for development and severity of pneumococcal infection is attributed to impairment of pulmonary host defences, favoring colonization and invasion of the upper and lower respiratory tract, respectively^4^. With the exception of our previous studies^5,6^, relatively little attention has, however, focused on the direct effects of smoking on the pathogen *per se*. These studies were the first to document that exposure of the pneumococcus to cigarette smoke condensate (CSC) *in vitro* results in genesis of biofilm^5,6^. Biofilm is a highly-hydrated viscoelastic extracellular matrix, comprised of various types of bacterium-derived polymeric materials, which not only insulates the pathogen against host defences but also obstructs penetration of antibiotics.

Resistance of the pneumococcus to macrolide antibiotics represents a problem of increasing magnitude as these agents are widely used in the treatment of pneumococcal infection^7^. As opposed to mutations in genes that confer antibiotic resistance, resistance of the pneumococcus to macrolides results from the horizontal transfer of two types of intact resistance genes. These are, firstly the gene encoding ribosomal methylase, which obstructs target binding, and secondly those encoding drug efflux pumps^7^.

Building on our previous studies on mechanisms of smoking-related antibiotic resistance and pneumococcal disease, the current investigation was undertaken to determine whether exposure to cigarette smoke condensate (CSC) of two strains of *S. pneumoniae*, harboring distinct genetic mechanisms of macrolide resistance, affected the expression of these antibiotic resistance genes. These were strains 2507 and 521 of the pathogen, both serotype 23F, commonly linked to invasive disease. The former expresses the *erm*(B) ribosomal methylase gene and the latter the macrolide efflux pump gene, respectively.

## METHODS

CSC from Murty Pharmaceuticals (Lexington, KY, USA) was dissolved to 40 g/L in dimethylsulfoxide (DMSO). The total amount of condensate^8^ generated during the combustion of a single cigarette is 26.3 mg, considerably higher than the concentrations of CSC used in the current study. Briefly, the two strains of the pneumococcus were grown overnight to mid-log growth phase in tryptone soy broth (TSB, Merck, Darmstadt, Germany) and optically standardized to 2.0×10^8^ colony-forming units (cfu)/ mL. The bacteria were then exposed to CSC (two successive 90 min exposures at final concentrations of 80 or 160 mg/L), followed by the addition of TSB or the macrolide antibiotic, clarithromycin (2 and 8 mg/L for strains 521 and 2507, respectively), for 15 min at 37^o^C, 5% CO_2_. These concentrations of CSC were based on our previous studies on pneumococcal biofilm formation^5,6^, while those of clarithromycin were based on minimum inhibitory concentration (MIC) values for strains 521 and 2507 of 2 and >256 mg/L, respectively^9^. DMSO solvent controls were included in all experiments. The bacterial cells were then concentrated by centrifugation, the pellet snap frozen in liquid nitrogen and stored at -80^o^C prior to undergoing a two-way RNA extraction process as described previously^6^.

Following RNA extraction, a high-capacity complementary DNA (cDNA) reverse transcription kit (Applied Biosystems, Foster City, CA, USA) was used according to the manufacturer’s instructions for the generation of cDNA.

Clarithromycin resistance gene expression was determined using real-time reverse transcription polymerase chain reaction (qRT-PCR) using Stratagene Brilliant II SYBR^®^ Green QPCR low ROX master mix (Agilent, Santa Clara, CA, USA) and PIKOREAL 96 well plates (ThermoScientific Inc.) on a PIKOREAL 96 real-time detection system (ThermoScientific Inc.), as described previously^6^. In the case of *erm*(B), the forward primer used was 5’– AGGGCATTTAACGACGAAAC–3’, while the reverse primer was 5’–GACGCATGGCTTTCAAAAAC–3’. The forward and reverse primers used for the antimicrobial resistance gene *mef*(A) were 5’–CTTTTCATACCCCAGCACTC–3’ and 5’– GCAATCACAGCACCCAATAC–3’, respectively. The housekeeping genes, *gyr*(A) and *gyr*(B) were also included.

Relative gene expression was performed^10^ by comparing the relative change in expression of the target genes to that of the reference genes and normalized to the untreated strains to reflect the log(10^-ΔΔCq^). The results are expressed as mean ± standard deviation (SD) of the log-fold increase of three different experiments with duplicate measurements for each system. Analysis of variance was measured using repeated-measures ANOVA with Tukey-Kramer multiple comparisons test as post-examination. Statistical significance was set at p<0.05.

## RESULTS

**Figure 1:**
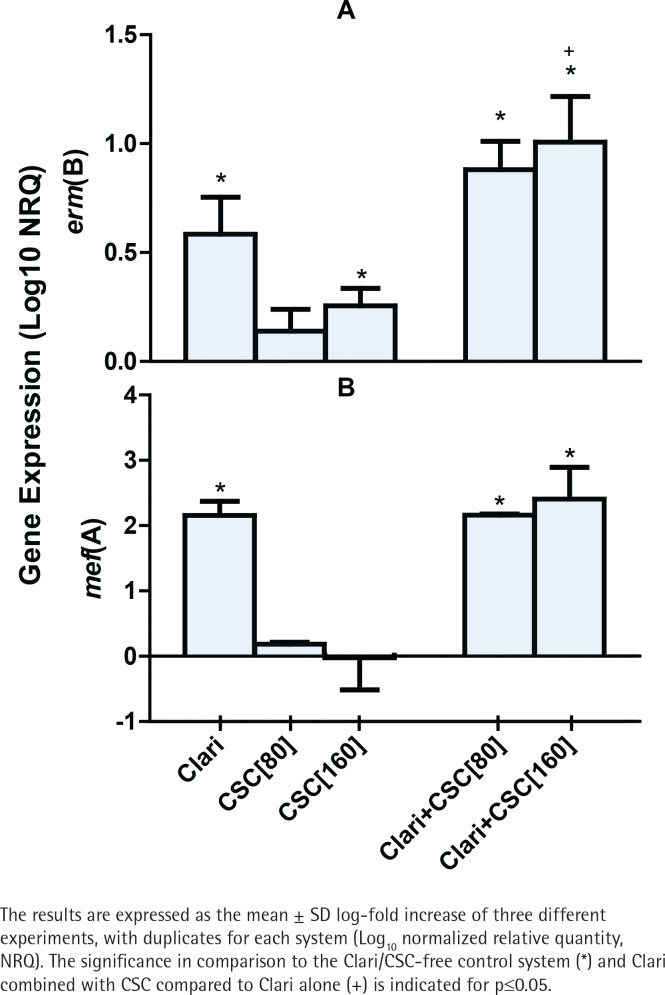
The effects of clarithromycin (Clari, 8 mg/L) and CSC (80 and 160 mg/L), alone and in combination, on the expression of erm(B) by strain 2507 (Figure 1A) and mef(A) by strain 521 (Figure 1B) of S. pneumoniae.

Expression of the *erm*(B) and *mef*(A) genes by strains 2507 and 521 of the pneumococcus, respectively, was undetectable in the absence of either CSC or clarithromycin. On the other hand, as shown in Figure 1A, exposure of strain 2507 of the pneumococcus to CSC alone, in the absence of clarithromycin, caused dose-related induction of the *erm*(B) macrolide resistance gene, which achieved statistical significance (p<0.05) at a concentration of 160 mg/L of the condensate. In addition, and as expected, exposure of strain 2507 of the pneumococcus to clarithromycin alone resulted in significant expression of the *erm*(B) gene, which was further and significantly (p<0.001) augmented by prior exposure of the pathogen to CSC at a concentration of 160 mg/L. The magnitude of gene expression for the system treated with the combination of clarithromycin and CSC at 160 mg/L was 27% higher (p<0.05) than that of the sum of the systems treated with the antibiotic and CSC individually, consistent with an augmentative interaction. Although not statistically significant, the corresponding increase for the system treated with the combination of clarithromycin and CSC at 80 mg/L was 25%.

In the case of strain 521, as shown in Figure 1B, expression of the *mef*(A) gene was significantly increased in the presence of clarithromycin alone, the magnitude of which was unaffected by prior exposure of the pathogen to CSC, while exposure to CSC alone had no detectable effect on gene expression. These differential effects of CSC on expression of the *erm*(B) and *mef*(A) genes may result from lack of effect of CSC on the transcriptional mechanisms involved in the induction of the *mef*(A) gene.

## DISCUSSION

### Gene Expression (Log10 NRQ) *erm*(B)

Our findings imply that smoking sensitizes macrolideresistant pneumococci in the airways for increased expression of the *erm*(B) gene. This proposed scenario is based on two observations. Firstly, that exposure of strain 2507 of the pneumococcus to CSC alone resulted in spontaneous induction of the *erm*(B) gene, a remarkable finding, which, to our knowledge has not been described previously. Secondly, that the magnitude of expression of the *erm*(B) gene following sequential exposure of the pneumococcus to CSC and clarithromycin was significantly greater than the sum of the individual exposures to CSC and the antibiotic.

These effects of CSC on both spontaneous induction and augmentation of clarithromycinmediated expression of the *erm*(B) gene may predispose to the development of a more aggressive resistance phenotype, characterized by a more rapid onset and greater magnitude of antibiotic resistance. This contention is based on a previous study, which demonstrated that following macrolide-mediated induction of the *erm*(B) gene, which happens rapidly, completion of the post-translational events is slow, with acquisition of the fully-resistant phenotype, only occurring after a lengthy lag period of up to twelve hours^11,12^. In the context of the findings of the current study, this lag period may be significantly shortened due to prior smoke-mediated induction of *erm*(B).

CSC-mediated induction of the *erm*(B) gene, is likely to be the consequence of a general stress response of the pneumococcus to CSC-mediated oxidative stress. In this context, cigarette smoke contains an abundance of pro-oxidative toxicants, including organic and inorganic highly-reactive free radicals and heavy metals, which trigger, directly or indirectly, the induction of various stress response genes to counter oxidative damage. Furthermore, exposure of the pneumococcus to CSC has previously been reported by us and others to cause significant upregulation of the two-component regulatory system 11 (TCS11), which is involved in the induction of genes associated with biofilm formation^6,13^, the efflux of various chemical and heavy metal toxicants, and, importantly in the context of the current study, those involved in promoting vancomycin resistance^10^. However, the transcriptional mechanisms, including possible involvement of TCS11, which mediate induction of the pneumococcal *erm*(B) gene, as well as the possible involvement of its product, ribosomal methylase, in attenuating oxidative stress remain to be identified. Nevertheless, it is noteworthy that ribosomal RNA methylation has been linked to protection against environmental/oxidative stress in both *Escherichia coli* and *Staphylococcus aureus* ^14,15^.

## CONCLUSIONS

**Figure 2:**
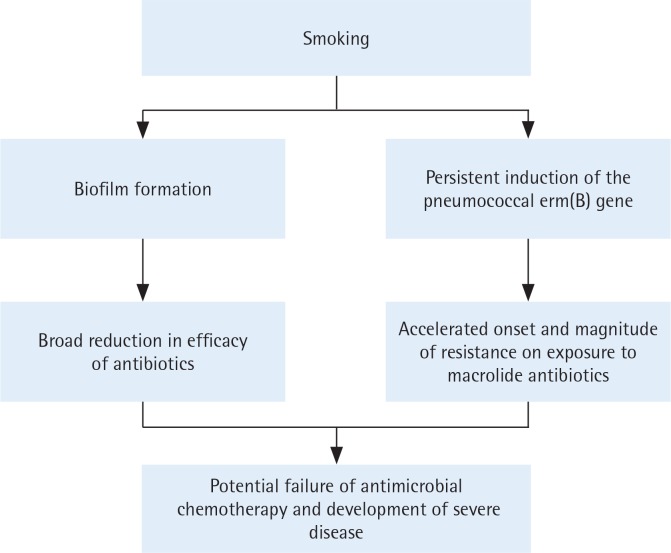
The mechanisms by which smoking may promote antibiotic resistance in the pneumococcus.

Based on the current and our previous studies, the mechanisms by which smoking may promote antibiotic resistance in the pneumococcus are summarized in Figure 2. Although the possible clinical significance remains to be established, these findings suggest that smoking may impede the efficacy of macrolidebased antimicrobial therapy by accelerating the onset and magnitude of *erm*(B)-mediated resistance, representing a novel pro-infective mechanism of smoking.

## References

[1] Cilloniz C, Martin-Loeches I, Garcia-Vidal C, San Jose A, Torres A (2016). Microbial etiology of pneumonia: epidemiology, diagnosis and resistance patterns. Int J Mol Sci.

[2] Nuorti JP, Butler JC, Farley MM (2000). Cigarette smoking and invasive pneumococcal disease. N Eng J Med.

[3] Bello S, Menéndez R, Antoni T (2014). Tobacco smoking increases the risk for death from pneumococcal pneumonia. Chest.

[4] Feldman C, Anderson R (2013). Cigarette smoking and mechanisms of susceptibility to infections of the respiratory tract and other organ systems. J Infect.

[5] Mutepe ND, Cockeran R, Steel HC (2013). Effects of cigarette smoke condensate on pneumococcal biofilm formation and pneumolysin. Eur Respir J.

[6] Cockeran R, Herbert JA, Mitchell TJ (2014). Exposure of a 23F serotype strain of *Streptococcus pneumoniae* to cigarette smoke condensate is associated with selective upregulation of genes encoding the two-component regulatory system 11 (TCS11). Biomed Res Int.

[7] Peyrani P, Mandell L, Torres A, Tillotson GS (2019). The burden of community-acquired bacterial pneumonia in the era of antibiotic resistance. Expert Rev Respir Med.

[8] Davies RF, Day TD (1969). A study of the comparative carcinogenicity of cigarette and cigar smoke condensate on mouse skin. Br J Cancer.

[9] Anderson R, Steel HC, Cockeran R (2007). Clarithromycin alone and in combination with ceftriaxone inhibits the production of pneumolysin by both macrolide-susceptible and macrolide-resistant strains of *Streptococcus pneumoniae*. J Antimicrob Chemother.

[10] Hellemans J, Mortier G, De Paepe A, Speleman F, Vandesompele J (2007). qBase relative quantification framework and software for management and automated analysis of real-time quantitative PCR data. Genome Biol.

[11] Wolter N, Smith AM, Farrell DJ, Northwood JB, Douthwaite S, Klugman KP (2008). Telithromycin resistance in Streptococcus pneumoniae is conferred by a deletion in the leader sequence of *erm*(B) that increases rRNA methylation. Antimicrob Agents Chemother.

[12] Cockeran R, Steel HC, Wolter N (2012). Effects of clarithromycin at sub-minimum inhibitory concentrations on early *erm*(B) gene expression, metabolic activity and growth of an *erm*(B)-expressing macrolide-resistant strain of *Streptococcus pneumoniae*. Open J Respir Dis.

[13] Li YH, Lau PC, Tang N, Svensäter G, Ellen RP, Cvitkovitch DG (2002). Novel two-component regulatory system involved in biofilm formation and acid resistance in *Streptococcus mutans*. J Bacteriol.

[14] Gómez-Mejia A, Gámez G, Hammerschmidt S (2018). *Streptococcus pneumoniae* two-component regulatory systems: The interplay of the pneumococcus with its environment. Int J Med Microbiol.

[15] Kyuma T, Kimura S, Hanada Y, Suzuki T, Sekimizu K, Kaito C (2015). Ribosomal RNA methyltransferases contribute to *Staphylococcus aureus* virulence. FEBS J.

